# A Successful Arthroscopic Management of a Benign but Locally Aggressive Tenosynovial Giant Cell Tumour in the Shoulder

**DOI:** 10.7759/cureus.57492

**Published:** 2024-04-02

**Authors:** Parthiban VJ, Anandanarayan Muruganandam, Suresh Perumal, Sanjay AK, Arumugam Sivaraman

**Affiliations:** 1 Arthroscopy and Sports Medicine, Sri Ramachandra Institute of Higher Education and Research, Chennai, IND

**Keywords:** challenges in arthroscopic synovectomy of shoulder, recurrence in pvns shoulder, tenosynovial giant cell tumour of shoulder, outcome in pvns shoulder, problems in arthroscopic extensile synovectomy, pvns shoulder

## Abstract

Introduction

Pigmented villonodular synovitis (PVNS) is a relatively rare disorder affecting the synovial membrane and tendon sheath of a joint. It rarely affects the shoulder joint. This prospective study aims to document the challenges encountered in achieving total synovectomy and assesses the clinical outcomes of arthroscopic synovectomy for PVNS in shoulder patients.

Methods

This is a prospective study conducted from April 2017 to September 2023. This monoarticular disease was observed among six patients (four females and two males). All patients underwent arthroscopic extensile synovectomy with biopsy and culture. The outcomes were measured using Constant score, American Shoulder and Elbow Surgeons (ASES), and University of California Los Angeles (UCLA) scores. All patients were followed up for a minimum of 36 months after arthroscopic synovectomy.

Results

All intraoperative findings were consistent with PVNS and confirmed with histopathological examination. All patients achieved a satisfactory, painless range of movements following surgery. The individual Constant score improved from a mean value of 64.83 to 94.50, the ASES score improved from a mean value of 81.15 to 99.73, and the UCLA score improved from a mean value of 23.16 to 34.83 post-arthroscopic intervention, proving its effectiveness. No recurrences were reported after 36 months of follow-up.

Conclusion

PVNS can be easily missed, and one must have a high index of suspicion to diagnose early. Delayed presentation of the disease had led to severe destruction of the joint. Early diagnosis and arthroscopic intervention prior to joint destruction are crucial for achieving a good functional outcome. Incomplete excision may lead to recurrence of the disease. Therefore, we propose extensile arthroscopic synovectomy of the shoulder, wherein by expecting and addressing the intraoperative challenges, complete excision can be achieved, thus preventing recurrence.

## Introduction

Pigmented villonodular synovitis (PVNS) is a relatively rare disorder affecting the synovial membrane and tendon sheath [1]. It is a type of tenosynovial giant cell neoplasm, generally considered benign with only local recurrence potential. There have been no reported cases of metastasis to date. PVNS exhibits a higher level of aggressiveness compared to other tenosynovial giant cell tumors. Typically, it affects the ankle, hip, and knee joints [2]. Recurrence rates range considerably, from 14% to 55%. Bertoni observed a 3% likelihood of malignant transformation following PVNS [3].

Only 2-8% of PVNS patients have shoulder involvement, making it quite uncommon [4]. Some case reports link PVNS of the shoulder with rotator cuff tears and chondral lesions [5]. Both genders are equally affected, with the mean age of diagnosis reported as 60.7 years for women and 43.5 years for men. The knee joint is the most commonly affected joint, followed by the hip joint. The shoulder, elbow, and ankle are a few other joints affected. Few studies suggest an association between PVNS and chronic recurring trauma. Treatment for PVNS has always involved either open or arthroscopic synovectomy. Until 2001, open synovectomy was the treatment of choice, but currently, with evidence from the literature, arthroscopic extensile synovectomy has become the gold standard treatment [6]. This prospective study aims to document the problems faced in achieving total synovectomy and also assess the clinical results of arthroscopic extensile synovectomy for PVNS patients.

We need to document the challenges faced during arthroscopic synovectomy of the shoulder, to help budding arthroscopic surgeons handle the unexpected difficulties intraoperatively, and to provide excellent care to the patients. One of the primary challenges encountered is the risk of removing normal tissues along with pathological tissues. Rotator cuff tears should always be investigated even if not suspected based on MRI findings. Loose bodies should be anticipated intraoperatively. Meticulous attention and care are essential to remove all pathological tissues completely, without leaving any remnants that could lead to local recurrence, and to achieve good functional outcomes.

## Materials and methods

Study design

This prospective study was carried out between April 2017 and September 2023 at Sri Ramachandra Institute of Higher Education and Research, Chennai, India.

Inclusion criteria

Patients presenting with complaints of either pain or restriction of shoulder movements and radiological investigations suggestive of PVNS were included in this study.

Exclusion criteria

Patients diagnosed with PVNS but treated with open surgical methods, patients who were not willing to participate in the study, and those lost to follow-up were excluded from this study. A total of six cases were included in this study after obtaining informed consent.

Clinical presentation and evaluation

**Figure 1:**
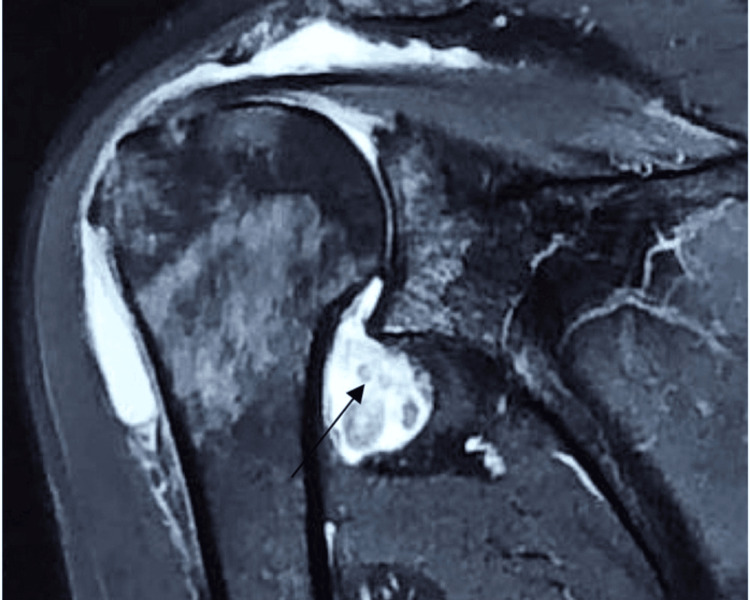
MRI demonstrating lobulated irregular intra-articular synovial thickening post-contrast enhancement

**Figure 2:**
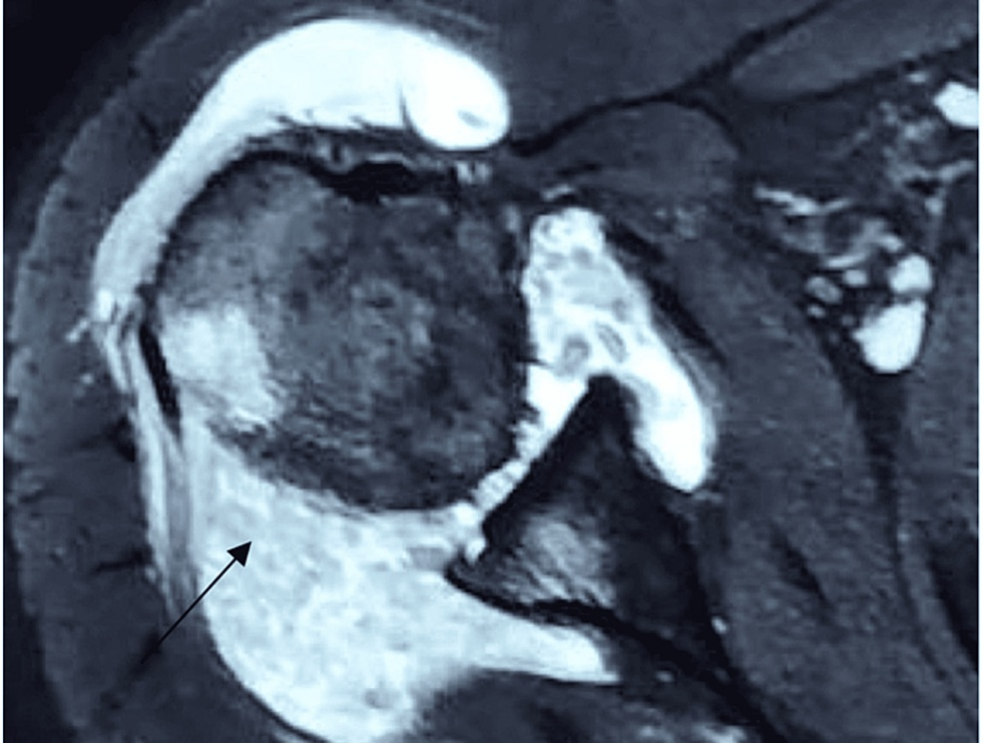
MRI revealing smooth erosion into the posterolateral aspect of the humeral head, indicative of pigmented villonodular synovitis

Patients typically presented with pain and swelling of the shoulder joint, with occasional locking and instability. X-rays showed increased soft tissue shadows and lower bone density around the shoulder. High signal intensity due to increased fat tissue was noted in magnetic resonance imaging (MRI), as shown in Figures 1-2. The diagnosis of PVNS was made based on tissue pathological reports. All biopsy tissues were examined for culture and sensitivity to detect infective organism growth.

Surgical technique

All arthroscopic procedures were conducted by the same surgical team. The patient was positioned in the lateral decubitus position with the arm suspended in the SPIDER-2 traction system (Smith and Nephew, Inc., Andover, USA). Following general anesthesia, two standard posterior and antero-inferior portals were established, and an arthroscopic examination was performed. Synovial proliferation tissues were removed using a grasper and shaver, and thorough debridement was performed in all cases. A drain was inserted and removed on postoperative day two.

Postoperative rehabilitation

The patient was immobilized with a broad arm sling postoperatively for one to two weeks. An active range of motion was encouraged as tolerated. Immediate post-procedure exercises could be performed.

Follow-up

All cases were followed up at the third, sixth, 12th, 24th, and 36th-month intervals. The average follow-up period was 36 months (about three years).

Outcome and statistical analysis

The outcome of this study was analyzed using various scores to measure pain, range of movement, and functional activity. These included the Constant score, University of California Los Angeles (UCLA) score, and American Shoulder and Elbow Surgeons (ASES) score. Scores obtained before the surgery were compared to those at the final follow-up. An increase in score at the final follow-up indicated an improved clinical outcome. An independent t-test was employed. A P-value of <0.05 was considered significant.

Data collection

Demographic and treatment data of patients included in this study were collected with appropriate permissions from the institute, and informed consent was obtained from patients for the same. Data were gathered from the medical records of the department of our institution, where the course of treatment was initiated and completed.

## Results

Patients information

The baseline information of the patients is shown in Table 1. Four of the patients were female and the other two were male. Their mean age was 50.66 years (range, 30-65 years). The mean body mass index (BMI) was 21.41 kg/m^2^ (range, 18.03-28.6 kg/m^2^). The average follow-up time was 36 months (range, 12-36 months).

**Table 1:** Baseline information of patients BMI: body mass index (kg/m^2^, where kg is a person's weight in kilograms and m^2^ is their height in metres squared. Overweight: 25.0 or more, healthy range: 18.5 to 24.9), DM: diabetes mellitus

Case No.	Age (in years)	Sex	Side	BMI	Comorbids	Complications
1	42	Female	Left	18.7	Type II DM	Nil
2	38	Female	Right	18	Nil	Nil
3	74	Male	Right	28.7	Type II DM	Nil
4	63	Female	Left	21.4	Systemic Hypertension	Nil
5	60	Female	Right	22.4	Nil	Nil
6	27	Male	Right	19.3	Nil	Nil

Operative findings

**Figure 3:**
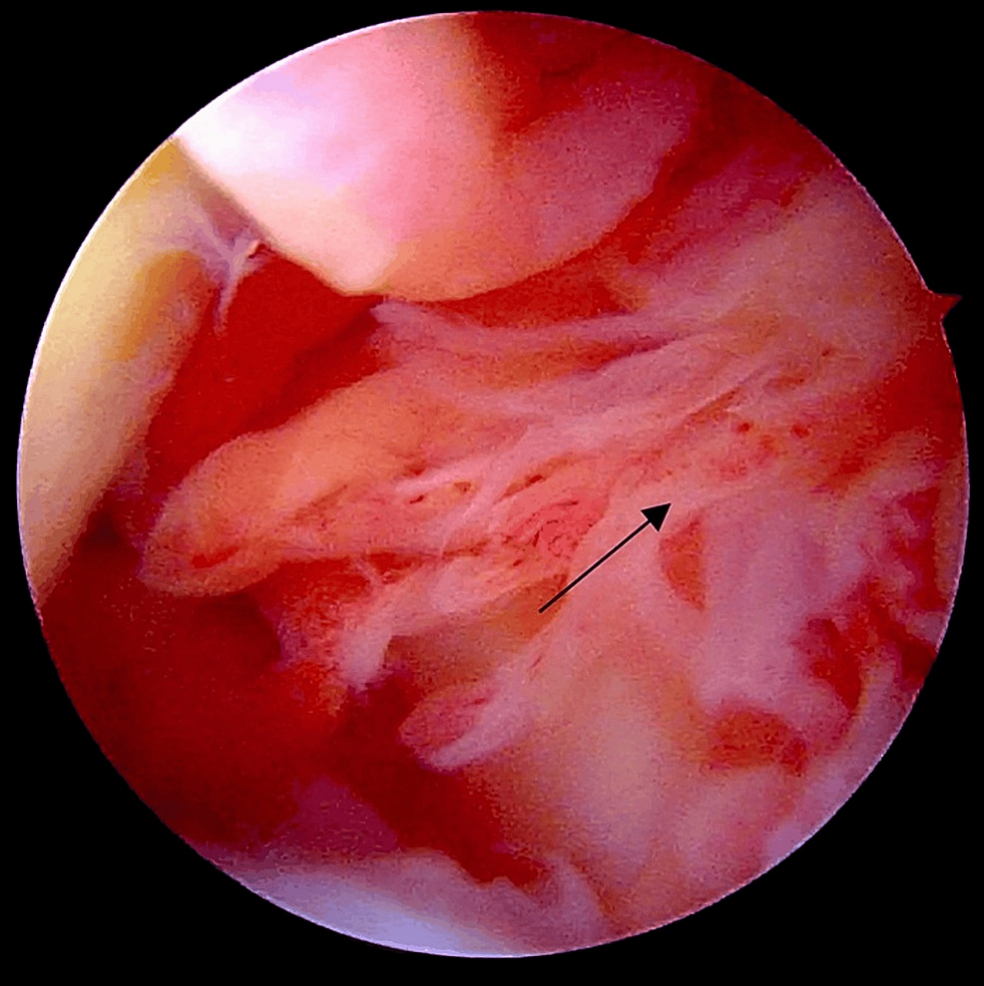
Intraoperative arthroscopic image displaying reddish-brown synovium with hyperplastic finger-like villous projections within the rotator interval

Intraoperatively, thick brownish synovial fluid and reddish-brown synovial hyperplastic tissue with finger-like projections were observed. More synovial growth was noted in the axillary pouch and rotator interval, as depicted in Figure 3.

Pathology

**Figure 4:**
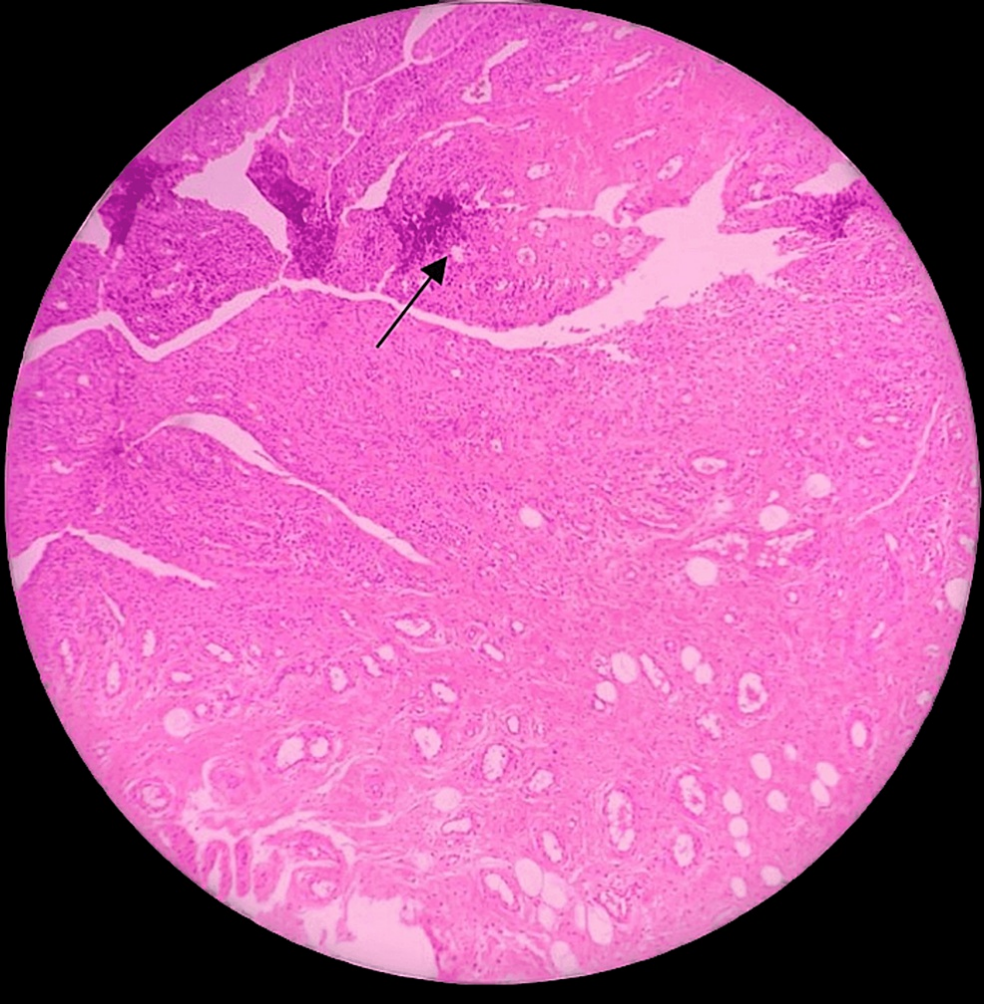
Displaying matted villi with thin-walled vascular channels

**Figure 5:**
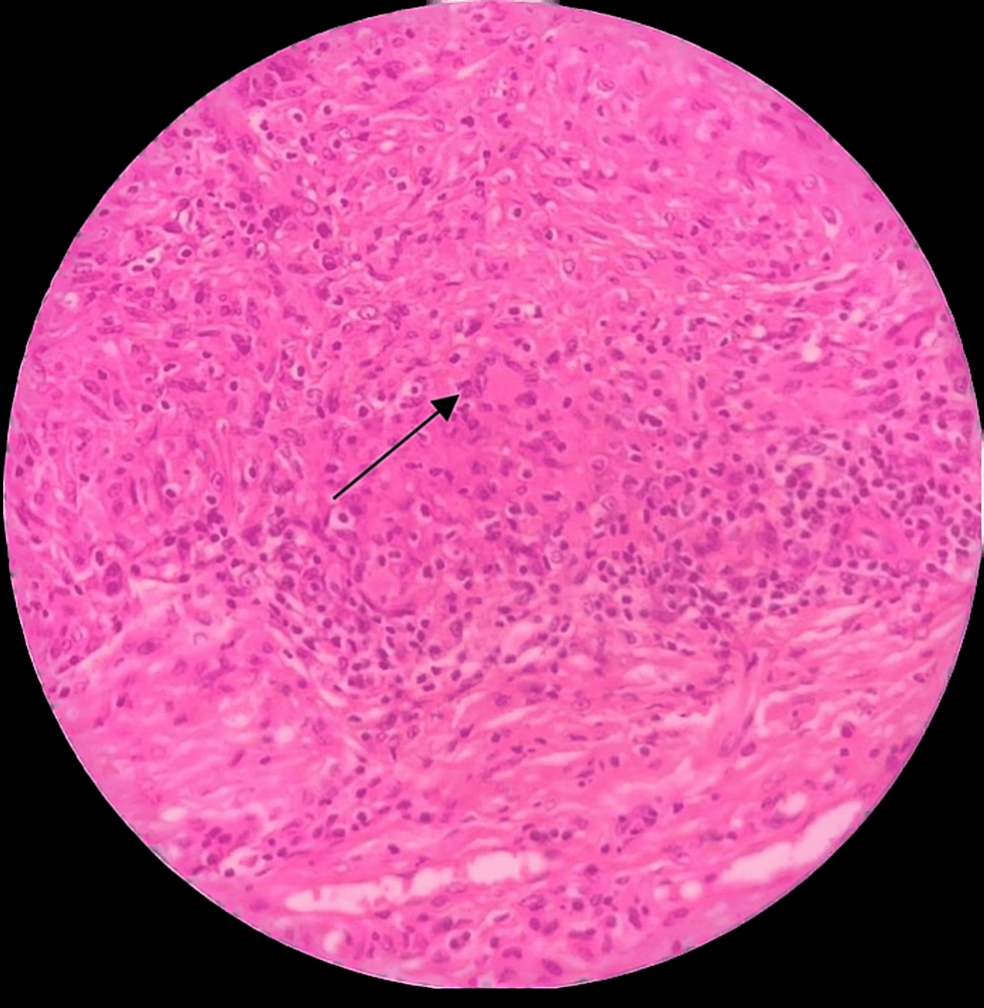
Depicting stroma packed with macrophages and multinucleate giant cells

Histopathologic specimens revealed synovial tissue with papillary projections and hyperplasia. Infiltration of monocytes, lymphocytes, and plasma cells was observed. The lesions consisted of matted villi with thin-walled vascular channels, as depicted in Figure 4. The stroma was filled with multinucleate giant cells, as shown in Figure 5. The diagnosis of PVNS was confirmed in all patients by the final pathological findings.

Functional score assessment

The functional scores were evaluated preoperatively and during the final follow-up visit. Patients showed statistically significant improvements in the CONSTANT, UCLA, and ASES scores during the final follow-up. Analysis of outcome by functional scores and statistical analysis of functional scores are shown in Tables 2-3, respectively.

**Table 2:** Analysis of outcome by functional scores ASES: American Shoulder and Elbow Surgery (maximum score: 100); Constant score (maximum score: 100); UCLA: University of California Los Angeles (maximum score: 35); Preop: Pre-operative; Postop: Post-operative; P value - <0.05 - Significant

Scores	Case 1	Case 2	Case 3	Case 4	Case 5	Case 6
Preop Constant score	57	68	86	56	70	52
Final follow-up Constant score	92	90	100	100	98	98
Preop ASES score	68.6	87.2	89.3	76.8	87	78
Final follow-up ASES score	98.4	100	100	100	100	100
Preop UCLA score	24	28	24	25	18	20
Final follow-up UCLA score	34	35	35	35	35	35

**Table 3:** Statistical analysis of functional scores ASES: American Shoulder and Elbow Surgery; UCLA: University of California Los Angeles; Preop: Pre-operative; Postop: Post-operative A P-value of <0.05 is considered to be significant.

Statistics	Constant score	ASES score	UCLA score
Preop Median value	62.5	82.5	24
Postop Median value	95.0	100	35
P-value of all cases	0.028	0.028	0.027

Challenges in achieving complete synovectomy

To attain a favorable functional outcome, meticulous attention and care are essential to remove all pathological tissues completely, without leaving any remnants that could lead to local recurrence. Injection of epinephrine into saline irrigation was used to maintain a clear field of vision and reduce bleeding [7].

Establishing proper working portals with needle guidance can be challenging due to increased bleeding. Identifying planes was difficult due to the quality and pathology of the tissues. Bleeding was encountered consistently due to the rich capillary supply of synovial projections. Achieving hemostasis was challenging and was accomplished with radiofrequency coagulation. A perioperative complication of saline pressure drop was resolved by employing a mechanical pump to regulate saline flow in a controlled manner. Turbulence control methods were routinely employed to manage saline flow and ensure a clear field of vision while using a shaver [8].

**Figure 6:**
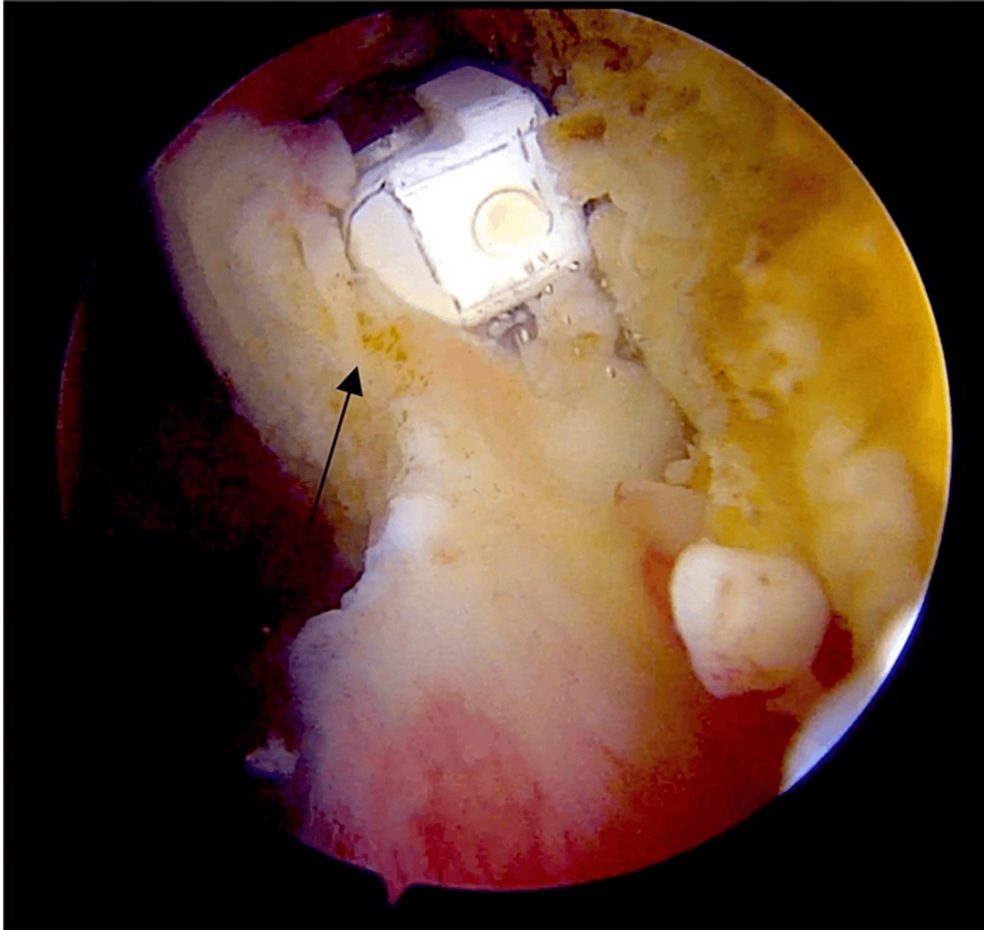
Depicting the demarcation of normal tissue engulfed with villous proliferation, meticulously debrided arthroscopically

One of the primary challenges encountered is the risk of removing normal tissues along with pathological tissues. Meticulous attention is required to remove only the pathological tissues to achieve thorough extensile synovectomy, as depicted in Figure 6, in order to prevent local recurrence. Rotator cuff tears should be investigated even if not suspected based on MRI findings. Loose bodies should be anticipated and systematically removed to ensure a complete synovectomy, starting from the subacromion, glenohumeral, anterior, and axillary recesses, followed by a thorough inspection for remnants after the synovectomy process.

Complications

The most anticipated complication, recurrence, was not encountered during the final follow-up period of this study. Intraoperative challenges included an obscured field of vision, difficulty in establishing the accessory portal, and the removal of pathological tissue adherent to normal tissue.

**Figure 7:**
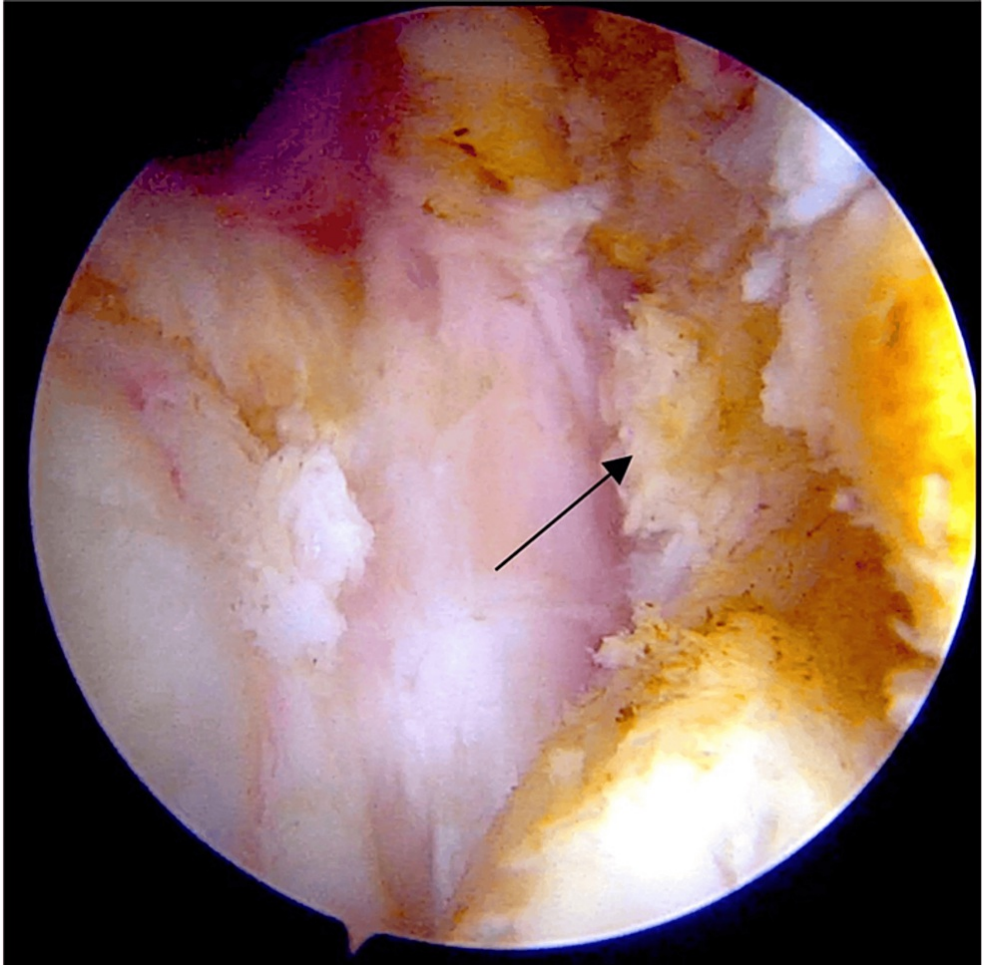
Showing intraoperative image of complete removal of pathological synovium by extensile arthroscopic synovectomy

Bleeding should be anticipated, and utmost care should be taken to perform a complete synovectomy. Incomplete synovectomy may lead to recurrence [9]. Some studies suggest a mean recurrence period of 4.5 years. Other potential complications include idiopathic numbness of the fingers [9]. This can be avoided by exercising meticulous attention at every step of the procedure to completely remove the inflamed synovium, as illustrated in Figure 7, at the conclusion of the extensile arthroscopic synovectomy.

## Discussion

Li et al. [9] observed unilateral involvement of PVNS in the shoulder, similar to our study, where participants presented with unilateral involvement. The etiology of PVNS still remains unknown. PVNS is categorized as a type of tenosynovial giant cell tumor, a rare tumor affecting the synovial membrane of the joints and tendon sheath, characterized by proliferation of the synovium [10].

Petsatodis et al. [11] observed two patients, both presenting in their 60s, but found equal involvement among both genders. In our study, six patients were observed, with the age distribution ranging between 27 and 74 years and equal distribution among both genders, consistent with the observations made by Petsatodis et al. [11]. Both genders are equally affected, with the mean age of diagnosis reported as 60.7 years for women and 43.5 years for men [11,12]. The knee joint is the most commonly affected joint, followed by the hip joint. PVNS is typically seen among older patients in a diffuse form [12]. Other joints affected include the ankle, shoulder, and elbow joints [13]. Myers et al. [13] suggested an association between PVNS and chronic recurring trauma. West et al. [14] proposed that translocation of chromosome 1p13 may be involved. Overexpression of colony-stimulating factor-1 (CSF-1) results in clusters of aberrant cell formation, leading to the creation of soft tissue hyperplasia of the synovium. Lipid metabolism disorder and chromosomal deviations have also been observed as causative factors by Cotten et al. [15].

PVNS manifests insidiously and progresses gradually. Patients typically present with chronic, intermittent joint pain, while some experience swelling and restricted movements even without pain [16]. Involvement of bilateral joints is uncommon. The symptoms observed in our study participants are similar to those reported by Byers et al. [16]. Rarely, knee joint effusion is observed in young patients, a symptom not typically seen in other joint involvements [17]. The most common symptoms noted among our study participants were pain, restricted range of motion, and swelling of the shoulder joint. Al Farii et al. [18] observed signs of soft tissue swelling and bone erosion on X-rays of the affected joints, findings similar to those in our study. Initial X-ray changes are often negative. Joint effusion, hemosiderin deposits, synovial expansion, and bone erosion were noted in the MRI of our patients, consistent with the findings of Xie et al. [19]. Diagnosis of PVNS typically takes about 18 months from the onset of symptoms [19]. On histopathological examination of synovial fluid, an assortment of mononuclear cells, extensive hemosiderin stores in macrophages, and multinucleated osteoclast-type giant cells are typically observed [20]. A definitive diagnosis can be made through the correlation of clinical, radiological, and histological findings. The histopathological examination of the patients in our study was similar to the findings observed by Frassica et al. [20], confirming the diagnosis of PVNS. Joint deformity, osteoarthritis, and degenerative articular changes are complications noted among patients who neglect treatment. Malignancy is suspected in cases of infiltration into both soft tissue and bone, although malignant changes are rare [21]. Some studies suggest that external beam radiation has been effective in reducing synovitis recurrence in patients undergoing synovectomy [21]. Recent advances, such as monoclonal antibodies and tyrosine kinase inhibitors, are under trial for controlling the overexpression of CSF-1 [22].

We acknowledge the limitations of this study. This is a prospective study with a small sample size from a single institution, and all the patients underwent the same procedure. However, PVNS of the shoulder is very uncommon, and based on our review of the literature, only 40 cases have been reported. Therefore, it is challenging to collect a large number of cases of patients who underwent the exact same procedure in a single institution. Additionally, this is not a comparative study, so we were unable to compare open and arthroscopic synovectomy. Furthermore, although radiotherapy is considered a safe and effective treatment for PVNS after incomplete resection [23], its effect was not included in this study.

## Conclusions

PVNS can be easily missed, and one must have a high index of suspicion to diagnose early. Investing time in detailed history and clinical examination and with imaging modalities such as MRI is essential for confirmation, planning, and documentation. Delayed presentation of the disease had led to severe destruction of the joint. Early diagnosis and arthroscopic intervention prior to joint destruction are crucial for achieving a good functional outcome.

Arthroscopic synovectomy is the gold standard treatment. However, incomplete excision may lead to recurrence of the disease. Therefore, we propose extensile arthroscopic synovectomy of the shoulder, wherein by expecting and addressing the intraoperative challenges, complete excision can be achieved, thus preventing recurrence.
